# Malaria, anaemia and under-nutrition: three frequently co-existing conditions among preschool children in rural Rwanda

**DOI:** 10.1186/s12936-015-0973-z

**Published:** 2015-11-05

**Authors:** Fredrick Kateera, Chantal M. Ingabire, Emmanuel Hakizimana, Parfait Kalinda, Petra F. Mens, Martin P. Grobusch, Leon Mutesa, Michèle van Vugt

**Affiliations:** Division of Internal Medicine, Department of Infectious Diseases, Centre of Tropical Medicine and Travel Medicine, Academic Medical Centre, Meibergdreef 9, 1100 DE Amsterdam, The Netherlands; Medical Research Centre Division, Rwanda Biomedical Centre, PO Box 7162, Kigali, Rwanda; Malaria and Other Parasitic Diseases Division, Rwanda Biomedical Centre, Kigali, Rwanda; Royal Tropical Institute, Koninklijk Instituut voor de Tropen, KIT Biomedical Research, Meibergdreef 39, 1105 AZ Amsterdam, The Netherlands; College of Medicine and Health Sciences, University of Rwanda, Kigali, Rwanda

**Keywords:** Malaria, Parasitaemia, Anaemia, Under-nutrition, Children, Rwanda

## Abstract

**Background:**

Malaria, anaemia and under-nutrition are three highly prevalent and frequently co-existing diseases that cause significant morbidity and mortality particularly among children aged less than 5 years. Currently, there is paucity of conclusive studies on the burden of and associations between malaria, anaemia and under-nutrition in Rwanda and comparable sub-Saharan and thus, this study measured the prevalence of malaria parasitaemia, anaemia and under-nutrition among preschool age children in a rural Rwandan setting and evaluated for interactions between and risk determinants for these three conditions.

**Methods:**

A cross-sectional household (HH) survey involving children aged 6–59 months was conducted. Data on malaria parasitaemia, haemoglobin densities, anthropometry, demographics, socioeconomic status (SES) and malaria prevention knowledge and practices were collected.

**Results:**

The prevalences of malaria parasitaemia and anaemia were 5.9 and 7.0 %, respectively, whilst the prevalence of stunting was 41.3 %. Malaria parasitaemia risk differed by age groups with odds ratio (OR) = 2.53; *P* = 0.04 for age group 24–35 months, OR = 3.5; *P* = 0.037 for age group 36–47 months, and OR = 3.03; *P* = 0.014 for age group 48–60 months, whilst a reduced risk was found among children living in high SES HHs (OR = 0.37; *P* = 0.029). Risk of anaemia was high among children aged ≥12 months, those with malaria parasitaemia (OR = 3.86; *P* ≤ 0.0001) and children living in HHs of lower SES. Overall, under-nutrition was not associated with malaria parasitaemia. Underweight was higher among males (OR = 1.444; *P* = 0.019) and children with anaemia (OR = 1.98; *P* = 0.004).

**Conclusions:**

In this study group, four in 10 and one in 10 children were found stunted and underweight, respectively, in an area of low malaria transmission. Under-nutrition was not associated with malaria risk. While the high prevalence of stunting requires urgent response, reductions in malaria parasitaemia and anaemia rates may require, in addition to scaled-up use of insecticide-treated bed nets and indoor residual insecticide spraying, improvements in HH SES and better housing to reduce risk of malaria.

## Background

Malaria, anaemia and under-nutrition are each associated with significant morbidity and mortality, particularly among children in sub-Saharan Africa [1–3]. Globally, malaria is responsible for over 450,000 deaths among children under 5 years [1]; anaemia is prevalent in 273 million (43 %) of children aged 6–59 months [2]; and severe under-nutrition affects about 20 million preschool-aged children living mostly in African and South-East Asia Regions [4]. In the majority of the affected children, all three conditions frequently co-exist and have been associated with long-term complications, including deficits in physical and cognitive development and poor school performance [5–8].

Anaemia is characterized by a reduction in haemoglobin concentration causing impairment in meeting the oxygen demands of the body. Anaemia results broadly from either ineffective erythropoiesis or increased loss of erythrocytes or both. The main causes of anaemia include acute or chronic blood loss, nutritional deficiencies (including vitamins A, B12, C and folic acid and iron) [9], infectious diseases [10–12] and genetic disorders [13, 14].

Malaria causes a substantial proportion of anaemia observed in malaria endemic settings [15, 16]. However, how much of the anaemia burden is associated with malaria, relative to other causes, and across the different strata of malaria endemicities has not been studied.

Studies elucidating associations between malaria and under-nutrition yield conflicting results [8]; with some suggesting that under-nutrition is associated with higher malaria morbidity and all-cause mortality outcomes [17–19], while others show no effect of under-nutrition on malaria [20]. Conversely, some studies have associated malaria with increased risk of under-nutrition [21] and *Plasmodiumfalciparum* infection has been associated with acute weight loss [22]. Additionally, improvements in growth and other anthropometric indexes have been described in children protected from malaria, by using both malaria chemoprophylaxis [23] and long-lasting insecticide-treated bed nets (LLINs) [24].

Given the extensive temporal and spatial correlation between malaria, anaemia and under-nutrition, any interaction (causal or increasing the likelihood of poor health outcomes on either diseases) may lead to synergistic deleterious effects on child health and development. Studies on interactions between malaria, anaemia and under-nutrition particularly among community preschool-aged children are few and inconclusive [15]. Most of these children carry these disease conditions in “hidden” pre-clinical stages and rarely present to medical personnel in the national health care system. This study measured the prevalence, investigated co-existence and assessed for risk determinants of malaria parasitaemia, anaemia and under-nutrition among preschool-going children in a rural Rwandan community.

## Methods

### Study site

**Fig. 1:**
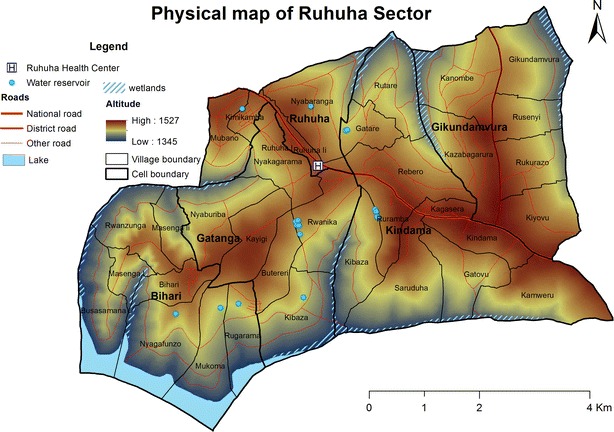
Map of Ruhuha sector, Bugesera district showing lay out of the 5 cells and associated key geographical features of elevation, wetlands, road net and a lake

Regarding administration, Rwanda has 30 districts: Each divided into sectors, cells, and villages locally term “umudugudus” (of about 50–100 households). This survey was conducted in 35 villages that are aggregated into five cells that constitute Ruhuha sector, Bugesera District in Eastern Rwanda (Fig. 1). Ruhuha sector is located 42 kms from Kigali city, has an area of 54 square meters and is separated from Burundi in the south by Lake Cyohoha. The sector has a population of ~23,900 individuals living in 5098 households (HHs): By sector, Gatanga has 1048 HHs, Ruhuha 696 HHs, Gikundamvura 869 HHs, Bihari 957 HHs and Kindama 1528 HHs. Ruhuha is a rural agricultural traditionally high malaria transmission setting with prior reported health facility slide positivity rates among sick individuals and community-based asymptomatic malaria positivity rates of 22 % and 5 %, respectively [25, 26].

### Study design and selection of study participants

A larger descriptive cross-sectional survey involving all study area HHs was conducted to study social, economic, entomological and biomedical determinants of residual asymptomatic malaria burden and transmission intensity. In summary, the night prior to the survey, a village community health care worker identified HHs to be visited from an enumeration list and requested heads of households (HoH) and family members to stay in-house. Upon providing consent, the study team (consisting of an interviewer and a laboratory technician) visited the notified HH and administered an interviewer-guided questionnaire to HoHs. In HHs where no member or no HoH or spouse was found present, a return visit was scheduled and attempted within 7 days. All HHs where the study team failed to conduct a survey on the return visit were excluded. Study findings from this larger sector-wide HH survey conducted between June and November 2013 have since been published [26]. For this sub-study, final data analysis was performed for only children aged 6–59 months who had complete laboratory and questionnaire data.

### Study procedures

#### Head of household interviews

A structured questionnaire was administered to the child’s primary caregiver to collect data on (1) demographics (sex, age, literacy, occupation, religion and marital status); (2) malaria prevention bed net (LLIN ownership, number and use) and indoor residual spraying (IRS) experience; (3) SES related variables (incomes, savings, land ownership, animals and sources of utilities like water, lighting and cooking) and HH structural features including type of outside wall, floor and roof materials); and (4) fever management practices. For each HH, location data was captured using a geographic positioning function based on the Samsung Galaxy 2 Android platforms (Samsung Electronics Co. Ltd, South Korea). The questionnaire used was written in English and was field-tested at three sites in order to minimize ambiguity, ensure consistency of comprehension of questions by both interviewers and respondents. Field workers were trained to administer the interviews in the local dialect (Kinyarwanda). Questionnaire data was collected using an electronic format developed using the open source Open Data Kit Collect setup on Android tablets [27].

#### Anthropometric measurements

Measures of under-nutrition indices (stunted, underweight, and wasted) were deduced from data on (1) age-in-months as reported by parents, (2) weight measured using UNICEF provided a digital Seca 874 weight scales (seca GmbH & Co. KG.) to the nearest 0.1 kg, and (3) height measured using a mobile measuring Seca 210 (seca GmbH & Co. KG.) mat for children 0 to 99 centimetres and a recumbent length board for children of height > 99 centimetres to the nearest centimetre, respectively.

#### Laboratory methods

From all HH members aged ≥6 months, finger-prick blood samples for malaria smear-based diagnosis and haemoglobin measurement were collected. Each smear was stained with 2 % Giemsa, processed and read independently by two study-trained microscopists at Ruhuha Health Centre laboratory. In case of a discrepancy, a tie-breaker third microscopist determined the final result. Expert microscopists at the National Reference Laboratory, Kigali, conducted quality control for all positive slides and a random sample of 5 % of all negative slides. Haemoglobin densities were measured on the spot in the field using a portable automated HemoCue^®^ Hb 301 haemoglobinometer system (HemoCue AB, Angelholm, Sweden) according to the product instruction.

#### Outcome and predictor variables

A blood smear was considered negative when light microscopy examination of 100 high-power fields did not reveal any asexual parasites and considered positive if any asexual parasites were detected on thick blood microscopy. In this study, malaria diagnosis was assessed based on presence of malaria parasites in blood by microscopy only. Data on reported symptoms or clinical signs was not collected. Anaemia was defined as moderate-to-severe haemoglobin levels of <90 g/L as recommended for disease surveillance, especially in areas of high anaemia prevalence [28]. Weight-for-height (wasting), height-for-age (stunting) and weight-for-age (underweight) *z* scores were calculated on the basis of the WHO Global Database on Child Growth and Malnutrition [29]. Z scores of <−2 SD were considered indicative of wasting, stunting, and underweight, respectively, while corresponding Z scores of <−3 SD were considered indicative of severe under-nutrition.

Predictor variables included HoH demographics (including age, sex, religion, marital status), cell of residence), malaria prevention practices (including availability and use of LLINs, HH use of IRS, reported prior fever management experiences, HH structure materials including type of floor (soil/clay/dung vs. brick/cement), roof (iron sheets vs. grass/tents), and outside walls (cement/brick versus mud/wood wall). A HH level SES/wealth index (used to categorize each HH as low, middle and high SES category) was generated using 10 indicator variables using principal component analysis [30].

#### Study consent and ethical approval

Written informed consent to participate in the study as well to allow study findings to be published in a relevant scientific journal was obtained from the HoH on behalf of all household members including minors. The National Health Research Committee (NHRC) and the Rwanda National Ethics Committee, Kigali, Rwanda (No 384/RNEC/2012) granted ethical and scientific approval for the study protocol.

### Statistical analysis

Data was collected using handheld android platforms on which open data kit software-hosted electronic questionnaire was loaded. These data was then relayed onto a server. Laboratory and anthropometric data was manually recorded in laboratory registers and later entered into Microsoft Access software. The two datasets were then merged and transferred into STATA 12.1 (STATA Corp., College Station, TX, USA) for analysis. Continuous variables were compared between groups (including stratification by age and sex) using Mann–Whitney U tests, and variable proportions were compared by Χ^2^ test. Associations between predictor variables and primary outcomes were statistically assessed for using both bivariate and multivariate logistic regression analysis. Odds ratios (ORs) and 95 % confidence intervals (CIs) were computed. Any covariate with a *p* value <0.15 in bivariate analysis were subsequently included in the final multivariable logistic model. Multi-collinearity tests were performed for all potentially correlated variables included in the final multivariate model for each of the five primary outcomes. Any risk estimate with a p-value <0.05 was considered statistically significant after adjustment for HH-level clustering and influence of other variables.

## Results

### Study population

As reported in the earlier publication for the larger survey, a total of 4705 HHs were surveyed and of these, data from 12,965 (all-ages) individuals from 3968 (84.3 %) HHs that had complete laboratory and questionnaire data was aggregated in the primary database [26]. From this primary database, data for 3182 children (aged 6–59 months) from 2228 HHs were extracted and analysed in this study. However for the final analysis, only 1882 (59.1 %) children with complete data on all primary outcome covariates (malaria slide positivity, haemoglobin densities and anthropometric data on age by months, height and weight) were considered.

Of the 1882 children, 50 % were female; the median age was 31.1 months (interquartile range (IQR), 18.4–45.7 months); the mean height was 85.8 cm and mean weight was 12.1 kgs. The median number of HH occupants was 5 (IQR 4–6) (Table 1). The prevalence of *P.falciparum* parasitaemia, moderate-to-severe anaemia and under-nutrition parameters of stunting, wasting and underweight were 5.9 %, 16.4 %, 41.3 %, 8.8 % and 15.8 %, respectively (Table 1).

**Table 1:** Study population baseline and demographic characteristics

Variable	Value (%) N = 1882
Sex	
Male	941 (50)
Female	941 (50)
Study group median age	31.1 (IQR: 18.4–45.70)
Study group mean weight in Kilograms	12.10 (±2.97)
Study group mean height in meters	85.93 ±12.35
Study group mean haemoglobin density in g/dl	11.30 (±1.53)
Number of children per cell	
Biharwe	296 (15.7)
Gatanga	474 (25.2)
Gikundamvura	372 (19.8)
Kindama	423 (22.5)
Ruhuha	317 (16.8)
Number of children with malaria parasite carriage (Yes)	110/1876 (5.9 %)
Number of children with moderate-severe anaemia (<90 g/l)	132/1876 (7.0 %)
Number of children with under weight (Z score of <−2 SD of mean)	297/1882 (15.8 %)
Number of children with stunting (Z score of <−2 SD of mean)	777/1882 (41.3 %)
Number of children with wasting (Z score of <−2 SD of mean)	166/1876 (8.8 %)
Number of children reported ownership of ≥1 Bednet in HH (Yes)	1799/1882 (95.6 %)
Number of HHs with reported IRS done in last 12 months (Yes)	1805/1882 (95.9 %)
Number of children with reported fever in last 6 months (Yes)	1293/1882 (68.7 %)
Number of HoH per education level None	620 (33 %)
Primary/Secondary/Tertiary	1259 (67 %)
Number of HH occupants	
1–3	298 (15.83 %)
4–7	1373 (72.95 %)
8+	211 (11.22 %)
Number of children per age group (in months)	
6–11	225 (11.96 %)
12–23	435 (23.11 %)
24–35	434 (23.06 %)
36–47	372 (19.77 %)
48–60	416 (22.10 %)

Plus–minus values are mean ± SD

*HH* households, *IQR* interquartile range

The proportions of children with co-morbidity were: 23/1882 (1.2 %) with malaria and anaemia; 59/1882 (3.1 %) with any under-nutrition and malaria 82/1882 (4.4 %) with any under-nutrition and anaemia. Only five children were found to have all three conditions concurrently (Table 2).

**Table 2:** Frequency of malaria, anaemia and under-nutrition co-morbidities

	Malaria, n (%)	Anaemia, n (%)	Under-nutrition, n (%)	Totals (n)
Malaria	38 (34.5)	23 (20.9)	59 (53.6)	110
Anaemia	23 (17.4)	27 (20.5)	82 (62.1)	132
Under-nutrition	59 (6.4)	82 (8.9)	782 (84.7)	923

The total number of children with malaria, anaemia and under-nutrition was 5

### Factors associated with malaria

Malaria prevalence differed by sex (7.5 % in females vs. 5.5 % in males: OR = 0.718; *P* = 0.034), age groups (compared to children aged <24 months, 24–35 months OR = 2.53; *P* = 0.04, 36–47 months age group OR = 3.5; *P* = 0.037, and for 48–60 age group, OR = 3.03; *P* = 0.014); and by cell of residence (10.9 % in Gikundamvura vs. 2.2 % in Ruhuha) (Table 3). By bivariate analysis, living in Gikundamvura (relative to living in Biharwe) cell was associated with a two-fold increase in odds of infection (P = 0.004). In the final multivariable model, malaria prevalence was significantly higher among older children (age-groups > 24 months), but was lower among children from high SES HHs (OR = 0.37; *P* = 0.029) (Table 4). A reduced malaria prevalence (borderline significant) was found among children; whose HH had used domestic water collected from a closed source (taps and boreholes) compared to HH where the used domestic water collected from an open source (OR = 0.62; *P* = 0.059), whose HoH had any education (OR = 0.63; *P* = 0.045); and whose house structure walls were made of bricks/cement (OR = 0.62; *P* = 0.061) (Table 4).

**Table 3:** Malaria parasitaemia and anaemia distributions and bivariate analysis stratified by sex, residence and age group

Variables	Malaria parasitaemia		Moderate–severe anaemia	
	N (%)	OR (95 % CI), P-value	N (%)	OR (95 % CI), P-value
Sex				
Male	49 (5.24)	0.796 (0.540–1.173), 0.248	70 (7.4)	1.139 (0.799–1.624), 0.470
Female	61 (6.49)	1	62 (6.6)	1
Age-group				
6–11	6 (2.68)	1	29 (12.9)	1
12–23	19 (4.37)	1.659 (0.653–4.216), 0.287	33 (7.6)	0.555 (0.327–0.940), 0.029
24–35	28 (6.51)	2.531 (1.032–6.206), 0.042	30 (6.9)	0.502 (0.293–0.860), 0.012
36–47	25 (6.74)	2.625 (1.059–6.502), 0.037	21 (5.6)	0.404 (0.225–0.728), 0.003
48–59	32 (7.69)	3.028 (1.246–7.356), 0.014	19 (4.6)	0.323 (0.177–0.591), <0.0001
Residence				
Biharwe	19 (6.42)	1	18 (6.1)	1
Gatanga	23 (4.85)	0.743 (0.398–1.390), 0.353	33 (6.9)	1.156 (0.638–2.092), 0.633
Gikundamvura	38 (10.30)	1.674 (0.943–2.969), 0.078	33 (8.9)	1.503 (0.829–2.728), 0.180
Kindama	23 (5.46)	0.843 (0.450–1.577), 0.592	26 (6.2)	1.011 (0.544–1.881), 0.971
Ruhuha	7 (2.22)	0.330 (0.137–0.798), 0.014	22 (6.9)	1.152 (0.605–2.193), 0.667

P values for categorical variables were based on the χ^2^ test

*OR* odds ratio, *CI* confidence interval

**Table 4:** Multivariate risk factors analysis for anaemia, under-nutrition parameters and malaria parasitaemia

Variable	Anaemia OR (95 % CI), P-value	Stunting OR (95 % CI), P-value	Wasting OR (95 % CI), P-value	Underweight OR (95 % CI), P-value	Malaria OR (95 % CI), P-value
Malaria parasitaemia (Positive)	3.857 (2.208–6.740), <0.0001				
Presence of anaemia (yes)				1.857 (1.093–3.155), 0.022	3.898 (2.297–6.615), <0.0001
Presence of stunting (yes)	1.428 (0.960–2.126), 0.079		0.046 (0.024–0.088), <0.0001	20.412 (12.304–33.862), <0.0001	
Presence of wasting (yes)		0.055 (0.030–0.098), <0.0001		59.139 (32.506–107.594), <0.0001	
Presence of under weight (yes)	1.979 (1.240–3.158), 0.004	20.256 (12.464–32.921), <0.0001	60.71 (31.994–115.197), <0.0001		
Sex					
Male				1.444 (1.061–1.966), 0.019	
Fever (Yes)		1.331 (1.070–1.656), 0.010			
Is HH in an economic group? (Yes)		0.758 (0.618–0.931), 0.008			
Was IRS done in HH (Yes)	0.471(0.222–0.997), 0.049		0.589 (0.397–0.873), 0.008		
Does the HH owns ≥1 bednet (Yes)		0.551 (0.338–0.899), 0.017			
Study participant age group					
6–11	–				–
12–23	0.522 (0.297–0.916), 0.023			1.262 (0.697–2.283), 0.443	1.989 (0.766–5.169), 0.158
24–35	0.405 (0.227–0.721), 0.002			1.849 (1.031–3.316), 0.039	3.157 (1.259–7.920), 0.014
36–47	0.340 (0.181–0.639), 0.001			2.124 (1.159–3.894), 0.015	3.528 (3.1384–8.992), 0.008
48–60	0.257 (0.135–0.490), <0.0001			2.123 (1.169–3.855), 0.013	3.699 (1.479–9.251), 0.005
HH SES level					
Low					
Middle	0.588 (0.373–0.928), 0.022				0.793 (0.415–1.516), 0.483
High	0.568 (0.352–0.916), 0.020				0.372 (0.152–0.906), 0.029
Does HH have a closed water source (Yes)			1.465 (0.969–2.214), 0.070		0.620 (0.377–1.018), 0.059
House wall material (Bricks and stones vs. wood/mud/tent)					0.615 (0.370–1.024), 0.061
House floor material (cement/concrete vs. mud/earth/dung)				0.447 (0.276–0.723), 0.001	
Highest HoH education level (None vs. any)					0.633 (0.404–0.990), 0.045

### Factors associated with anaemia

Anaemia distribution was similar across both sexes but decreased with increasing age and, similar to malaria parasitaemia, showed a higher proportion among children in Gikundamvura cell (10.2 %) vs. children in the other four cells with proportions ranging from 5.4 to 6.3 % (Table 3). By bivariate analysis, living in Gikundamvura was associated with a 1.9-fold (*P* = 0.008) higher odds of having anaemia compared to living in Biharwe cell while the risk of anaemia decreased with increasing age group (Table 3). In the final multivariate model, anaemia risk was high among children with malaria infection (OR = 3.86) and underweight (OR = 1.98) and decreased with increasing age, and among children living in wealthier HHs of middle and high SES (OR = 0.59; *P* = 0.022 and OR = 0.57; *P* = 0.020, respectively) (Table 4).

### Factors associated with under-nutrition

Under-nutrition parameters showed varying co-existence patterns (Table 5). In summary, (1) underweight was associated with stunting (OR = 20.41; *P* ≤ 0.0001) and wasting (OR 59.14; *P* ≤ 0.0001); (2) stunting was associated with underweight (OR = 20.26; *P* ≤ 0.0001) but not wasting (OR = 0.06; *P* ≤ 0.0001); and (3) wasting was associated with underweight (OR = 60.71; P ≤ 0.0001) but not stunting (OR = 0.05; *P* ≤ 0.0001). In the final multivariate model, other predictors of (1) stunting were a reported fever history (OR = 1.33; *P* = 0.01); living in a house where the HoH belonged to a higher economic group (OR = 0.79; *P* = 008) and living in HHs that has a reported ownership of ≥1 LLIN (OR = 0.55; *P* = 0.017) (2) wasting were reported IRS applied in the HH (OR = 0.59; *P* = 0.008), and domestic water source (HHs using closed source had OR = 1.47; *P* = 0.07) and (3) underweight were sex (male 0R = 1.44; *P* = 0.019), age group, house floor material (bricks/cement OR = 0.45; *P* = 0. 001).

**Table 5:** Stunting, under-weight and wasting distributions and bivariate analysis stratified by sex, residence and age group

Variables	Stunting		Under weight		Wasting	
	N (%)	OR (95 % CI), P-value	N (%)	OR (95 % CI), P-value	N (%)	OR (95 % CI), P-value
Sex						
Male	404 (42.93)	1.146 (0.953–1.377), 0.147	167 (17.75)	1.346 (1.049–1.727), 0.020	79 (8.41)	0.897 (0.652–1.235), 0.506
Female	373 (39.64)	1	130 (13.82)	1	87 (9.28)	1
Age-group						
6–11	84 (37.33)	1	25 (11.11)	1	22 (9.78)	1
12–23	191 (43.91)	1.314 (0.945–1.827), 0.105	59 (13.56)	1.255 (0.763–2.066), 0.371	41 (9.45)	0.963 (0.558–1.660), 0.891
24–35	193 (44.47)	1.344 (0.967–1.869), 0.079	78 (17.97)	1.753 (1.082–2.840), 0.023	38 (8.84)	0.894 (0.515–1.553), 0.692
36–47	146 (39.25)	1.084 (0.771–1.525), 0.641	65 (17.47)	1.694 (1.033–2.777), 0.037	32 (8.63)	0.871 (0.493–1.540), 0.635
48–59	163 (39.18)	1.081 (0.774–1.511), 0.646	70 (16.83)	1.618 (0.993–2.639), 0.053	33 (7.93)	0.795 (0.451–1.399), 0.427
Residence						
Biharwe	114 (38.51)	1	52 (17.57)	1	27 (9.12)	1
Gatanga	217 (45.78)	1.348 (1.003–1.812), 0.048	83 (17.51)	0.996 (0.680–1.459), 0.984	38 (8.03	0.870 (0.519–1.458), 0.598
Gikundamvura	148 (39.78)	1.055 (0.771–1.442), 0.738	64 (17.20)	0.975 (0.652–1.458), 0.902	35 (9.46)	1.041 (0.614–1.763), 0.881
Kindama	160 (37.83)	0.971 (0.715–1.318), 0.852	58 (13.71)	0.746 (0.496–1.121), 0.158	38 (9.0)	0.986 (0.588–1.654), 0.957
Ruhuha	138 (43.53)	1.231 (0.891–1.699), 0.207	40 (12.62)	0.678 (0.433–1.059), 0.088	28 (8.89)	0.972 (0.558–1.692), 0.920

P values for categorical variables were based on the χ^2^ test

*OR* odds ratio, *CI* confidence interval

Collinearity analysis between all variables included in each of final multivariate model for the five primary outcomes showed mean variance inflation factor (VIF) values ranging from 2.17 to 7.23. For each of the five-outcome models, no variable showed a VIF >10: This is the cutoff marker of multicolinearity.

## Discussion

At least 4/10 and 1/10 preschool-age children in this rural setting were found stunted and underweight, respectively. Observed malaria parasite rates of <10 % suggest that this area is hypo-endemic for malaria. In this study, the proportion of children aged under 5 years with malaria (6.5 %) was almost two-fold higher than the 3.4 % reported in the same province in 2010 [31]. These differences in proportions infected with malaria may be partially explained by malaria-associated temporal patterns, seasonality and/or differences in sampling technique used in the two surveys. However, the parasite rates observed in this study are comparable to the <10 % malaria infection rates reported among community members previously [25, 26, 31] suggesting that this area is of hypo-endemic transmission intensity [26].

In this study, risk of malaria increased with increasing age. Findings in this study are consistent with an increasingly observed trend of higher malaria risk among older age groups, as reported previously elsewhere and in this area, following the scale-up of control interventions [25, 26, 32, 33]. A reduction in malaria transmission and hence a lower frequency of exposure to malaria parasite inoculation and the associated infections impedes and, plausibly, delays development of a malaria protective immunity leading to an increased risk of malaria in older age groups. However, two reasons may account for the higher risk in older children observed in this study: (1) younger children are more likely to sleep under ITNs and hence be more protected, and (2) in contrast, older children are likely to tolerate malaria parasites without developing a fever and hence have an increased prevalence of asymptomatic malaria parasitaemia.

In this study, a protective malaria risk was associated with living in a high SES HHs. However, studies on associations between malaria and SES have hitherto yielded conflicting results, with some indicating no associated effect [34, 35] while others have shown that higher SES induces a protective effect [36–40]. It is plausible that a protective effect may exist where improved house structural features lead to a reduction in indoor malaria transmission by restricting mosquito entry [40]. Individuals living in houses whose wall structure were made of wood/mud (*vs* cement/bricks) showed a significantly higher risk of malaria in this study. Houses whose walls are made of mud have been associated with having more eaves (that support mosquito entry), higher risk of indoor mosquito bites and, by creating cooler and darker conditions in comparison to brick/cement houses, creating a favourable indoor resting environment for mosquitoes [41–43].

The proportion of children under 5 years found with anaemia in this study (6.8 %) was three-fold higher than the 2.0 % reported for the same province in 2010 [31]. After adjusting for all predictors, anaemia risk was associated with malaria parasitaemia, age group, HH SES and underweight, with a borderline significant outcome noted among children with stunting and those coming from HHs where IRS was applied. Individuals found with moderate-to-severe anaemia had an almost four-fold increased risk of being malaria-infected. In malaria-endemic settings, malaria is the most common cause of anaemia [10] and among parasitaemic patients in comparable settings, a similarly increased risk of anaemia has been previously demonstrated [35, 44, 45]. In one study among preschool-going children in Uganda, malaria was found to be the only risk determinant for anaemia [16]. Additionally, effective malaria control programmes have been shown to significantly reduce anaemia burden, with anaemia now considered a surrogate indicator of impact of malaria control programmes [46, 47]. Interestingly in this study, the risk of anaemia decreased with increasing age groups in contrast to the observed increasing risk of malaria across the same age groups in our study and some studies other malaria-endemic settings [15, 35, 48]. However, in all the other settings, the reported malaria parasite carriage rates were significantly higher than the 6.8 % reported in this study. In study areas of lower parasitaemia carriage, and especially following reduction in malaria burden, malaria may make a less significant attributable contribution to anaemia relative to other risk factors [46].

Anaemia, but not malaria, was significantly associated with underweight in this study. Evidence for the impact of under-nutrition on development of anaemia in young children living in malaria-endemic areas had been reported previously [9]. Previous studies on malaria and under-nutrition associations have shown contrasting results. In Ghana and in The Gambia, under-nutrition was associated with increased risks of malaria-associated mortality and the risk of having multiple malaria episodes, respectively [17, 18]. In contrast, in Burkina Faso and Uganda, no association between under-nutrition and malaria morbidity was demonstrated [35, 49]. Although this study did not assess for other causal factors associated with anaemia, it is plausible that children who are malnourished are more likely to also have had micronutrient deficiencies that may have partly contributed to the burden of anaemia observed.

Children from middle and high SES HHs were found to have a significantly reduced risk of having anaemia than children from low SES HHs. Two plausible reasons for this are the differential nutritional intakes and house structural features that determine risk of indoor malaria transmission between the two SES levels. Presumably, children from low SES HHs are likely to have poorer nutritional intake and also live in houses whose structure are more conducive for indoor malaria transmission, with the increased risk of malaria causing a concurrent deleterious effect on anaemia risk.

Among preschool-aged children in the same province in 2010, stunting, wasting and underweight proportions in comparison to findings in this study were 43.9 %, 3.2 % and 11.5 % vs. 41.6 %, 8.8 % and 15.8 %, respectively [31]. Both surveys point to very high prevalence of stunting in this age group in this area. In our study, male sex was associated with a 1.44-fold increase in risk of underweight. Sex differences in risk of under-nutrition have been shown elsewhere [50, 51], but studies elucidating the observed sex-dependent risk of under-nutrition are lacking. As reported previously, the risk of being underweight significantly increased with increasing age in this study [52, 53]. Possible reasons for increased risk of underweight with increasing age could include but are not limited to (1) short birth intervals with mothers not having adequate gestational weight gains and hence having smaller than expected babies at birth; (2) reduced breast feeding periods; (3) poor weaning diets and; (4) reduced care given to older children following successive births [53].

Other risk determinants for malaria, anaemia and under-nutrition metrics were also identified. Living in houses where the HoH was not educated and in houses where domestic water was sourced from an open source compared to HHs where domestic water was drawn a closed source were associated with a high risk of malaria infection. Lack of education may likely be associated with low SES status and limited malaria control-associated knowledge and practice factors, which may be related to lower availability and use of malaria control measures like LLINs. Regarding the domestic water sources, open water sources may also serve as potential mosquito breeding sites and hence pose an increased risk. Anaemia risk was interestingly found to be lower by almost 50 % among individuals living in HHs where IRS was carried out. It is plausible that the IRS effect on lower anaemia risk was mediated primariry through reducing malaria risk. However, other unmeasured risk determinants may have contributed to the high risk among individuals from HHs where no IRS was applied of anaemia. Other identified risk determinants for under-nutrition included SES indicator variables (HoH belonging to an economic group and type of material house floor is made of) and prior fever experience and malaria control measures (LLIN availability and IRS experience). Given that the study area is predominantly agricultural, HHs where the HoHs reported being a member of an economic group are more likely to have better food security and hence a lower risk of under-nutrition. Individuals who reported having had a fever during the past 6 months are likely to have had malaria: A risk determinant of stunting [18]. The use of malaria control measures (LLIN and IRS) could have reduced the risk of malaria and limited long-term development of under-nutrition.

This study had several limitations. Being a cross-sectional study design, associations observed may have been confounded by unmeasured factors. Also, causal inferences cannot be drawn from study findings due to the study design employed. The lack of additional haematological assessments including mean cell volume, micronutrients and haemolysis parameters limited the characterization of anaemia types. With regard to under-nutrition, only weight and height measurements were collected. The lack of other under-nutrition related data including skin fold thickness, oedema and body mass index did not allow for a more robust nutritional assessment. In this survey, statistical adjustments for correlation with and between HH members were conducted to ensure study finding were robust. However, given the multi-factorial causal factors associated with anaemia and under-nutrition, data on important covariates including but not limited to helminthic infection, micronutrient levels, co-infections like HIV, genetic haemoglobin disorders and breast feeding need to be collected in future studies to be able to perform a more robust analysis.

## Conclusion

Study findings pointed to high rates of under-nutrition and anaemia but not malaria parasitaemia in preschool-going children. A strong association between malaria and anaemia but not between malaria and under-nutrition was observed. Although the study design limits the interpretation of cause and effect between these three disease determinants, control of malaria may have a substantial indirect reduction on anaemia burden among preschool-going children in this area. Integrated rather than vertical programmes covering nutritional rehabilitation, malaria control including the scaled up LLIN and IRS coverage, improvements in HH SES and better housing that limits mosquito entry are need to realize optimal child health outputs.
